# Brain Network for the Core Deficits of Semantic Dementia: A Neural Network Connectivity-Behavior Mapping Study

**DOI:** 10.3389/fnhum.2017.00267

**Published:** 2017-05-19

**Authors:** Yan Chen, Keliang Chen, Junhua Ding, Yumei Zhang, Qing Yang, Yingru Lv, Qihao Guo, Zaizhu Han

**Affiliations:** ^1^State Key Laboratory of Cognitive Neuroscience and Learning and IDG/McGovern Institute for Brain Research, Beijing Normal UniversityBeijing, China; ^2^Department of Neurology, Huashan Hospital, Fudan UniversityShanghai, China; ^3^Department of Neurology, Beijing Tiantan Hospital, Capital Medical UniversityBeijing, China; ^4^Department of Radiology, Huashan Hospital, Fudan UniversityShanghai, China

**Keywords:** semantic dementia, critical region, brain network, left anterior hippocampus, semantic deficits, graph theory

## Abstract

Individuals with semantic dementia (SD) typically suffer from selective semantic deficits due to degenerative brain atrophy. Although some brain regions have been found to be correlated with the semantic impairments of SD patients, it is unclear if the damage is actually responsible for SD patients’ semantic disorders because these findings were primarily obtained by examining the roles of local individual regions themselves without considering the influence of other regions that are functionally or structurally connected to the local individual regions. To resolve this problem, we investigated, from the brain network perspective, the relationship between the brain-network measures of regions and connections with semantic performance in 17 SD patients. We found that the severity of semantic deficits of SD patients was significantly correlated with the degree centrality values of the left anterior hippocampus (aHIP). Moreover, the semantic performance of the patients was also significantly correlated with the strength of gray matter functional connectivity of this region and two other regions: the left temporal pole/insula (TP/INS) and the left middle temporal gyrus. We further observed that the strength of the white matter structural connectivity of the left aHIP-left TP/INS tract could effectively predict the semantic performance of SD patients. When we controlled for a wide range of potential confounding factors (e.g., total gray matter volume), the above effects still held well. These findings revealed the critical brain network with the left aHIP as the center that could be contributing to the semantic impairments of SD.

## Introduction

Semantic dementia (SD) is a neurodegenerative disease that is characterized by a core symptom of selective semantic deficits (Mesulam et al., 2012) with progressive cerebral atrophy (Hodges et al., 1992; Mummery et al., 2000). It is clinically and theoretically important to reveal the neural network associated with the semantic deficits of SD, i.e., the SD-semantic network (Hodges et al., 1992; Mummery et al., 2000; Patterson et al., 2007). Studies have found that the atrophy of certain brain regions (e.g., the temporal pole, hippocampus, fusiform gyrus, superior temporal gyrus) are correlated with the semantic disorders of SD (Snowden et al., 1989; Galton et al., 2001; Desgranges et al., 2007; Ding et al., 2016). However, these findings were primarily obtained by examining the roles of the local individual regions themselves without considering the influence of other regions that functionally or structurally connect with the local individual regions. Indeed, the encephalatrophy of SD attacks a pre-existing network of brain regions that are interconnected (Seeley et al., 2009; Zhou et al., 2012; Guo et al., 2013; La Joie et al., 2014; Huth et al., 2016). Hence, the semantic deterioration of SD might originate from the linked disconnections and regional dysfunctions in the brain network (Wernicke, 1977). In other words, the observed effects of the local individual regions may be driven by other regions and/or connections with those regions. To find the regions and connections that contribute to SD-semantic deficits, it is necessary to evaluate the importance of a region or a connection to the semantic processing of SD from a brain-network perspective.

Graph-theoretical network analysis has been recently applied to depict the topological attributes of a large-scale brain network (Bullmore and Sporns, 2009). One of the most widely used indices in a graph-theoretical network analysis is degree centrality, which is measured as the number and/or strength of functional connections (FC) from a given region to all other regions in the network. The measure reflects the importance and centrality of a region in the network. Recently, Agosta et al. (2014) performed a sophisticated study and found that SD patients had a decreased degree centrality value of the inferior temporal and occipital cortices. Further issues that must be explored include (1) which regions with decreased degree centrality are correlated with semantic deficits of SD patients; (2) which FCs connecting with the core regions are responsible for the semantic deficits; and (3) if the relevant FCs have anatomical bases.

To provide empirical evidence for the aforementioned questions, the present study investigated the relationship of the semantic performance with brain network measures in 17 SD patients. We collected three types of images on the subjects (structural T1 MRI, resting-state functional MRI and diffusion-weighted images). We first identified the SD-semantic-related regions whose decreased functional degree centrality values were correlated with semantic performance of SD patients. Then, we performed the correlation analyses between the semantic performance and the strengths of functional and structural connectivity of the SD-semantic-related regions. The observed effects were also verified by factoring out the influence of multiple potential confounding factors (e.g., total gray matter volume).

## Materials and Methods

### Participants

Seventeen patients were selected from a cohort of SD patients (Ding et al., 2016). Each of our patients met the diagnostic criteria of SD proposed by Gorno-Tempini et al. (2011). A SD patient should exhibit all the following core diagnostic features:

(1) Impaired confrontation naming (which was measured by two tasks: oral picture naming and oral sound naming; Fang et al., 2015; Ding et al., 2016).
(2) Impaired single-word comprehension (measured by three tasks: picture associative matching, word associative matching and word-picture verification; Han et al., 2013; Bi et al., 2015; Fang et al., 2015; Ding et al., 2016).
(3) Predominant anterior temporal atrophy (measured by the gray matter volume in anterior temporal lobe).

Moreover, the patient should also present at least 3 of the following other diagnostic features:

(1) Surface dyslexia (measured by correct numbers on regular words minus those on irregular words and measured by regularization errors in word reading task; Ding et al., 2016).
(2) Impaired object knowledge, particularly for low-frequency or low-familiarity items (measured by naming to definition task; Ding et al., 2016).
(3) Spared repetition (measured by oral repetition task, Han et al., 2013; Ding et al., 2016).
(4) Spared speech production (measured by percentage of reasonable sentences for Cookie Theft task; Goodglass and Kaplan, 1972).

An impaired or spared performance of patient in an above task was defined by the corrected *t*-score in the task lower or higher than -1.96, respectively. The method calculating the corrected *t*-score was described in the Section “Behavioral Data Preprocessing” below. Predominant anterior temporal atrophy was defined by gray matter volume of anterior temporal lobe (comprising 83, 84, 87, and 88 subregions in AAL atlas) lower than two standard deviations below the average of the healthy control cohort of Ding et al. (2016).

Two of the 19 subjects reported in Ding et al. (2016) were excluded due to visible stripes in resting-state functional connectivity maps which might be caused by image artifacts. To increase the number of observations and the variations of behavioral and imaging data, three patients were tested more than once (two patients: two times; one patient: three times). The time interval between two successive observations of one patient was greater than 300 days (range: 340–408 days) to ensure independency. Thus, we collected 21 observations in total. The observations corresponded to 10 males and 11 females with mean age of 61.57 years (standard deviation = 8.77 years old). Our analyses were primarily performed on the 21 observations and later considered the confounding effects of observation times on each patient. We also chose 18 healthy controls (seven males, 66.44 ± 4.09 years old). The two groups revealed no significant difference in age, gender distribution, or education level (*p-*values >0.40; **Table 1**). This study was approved by the Institutional Review Board of the Huashan Hospital Affiliated with Fudan University. All subjects were right-handed, native Chinese speakers and provided written informed consent. This study was performed in accordance with the Revised World Medical Association’s Declaration of Helsinki.

**Table 1:** Demographic and neuropsychological profiles of SD patients and healthy control subjects.

	SD patients (*n* = 21)^†^	Healthy controls (*n* = 18)	Group difference (*t values or χ^2^ values*)
**Background information**			
Age (years)	61.57 (8.77) ^‡^	60.44 (4.09)	0.53
Gender (M/F)	10/11	7/11	0.67
Education (years)	11.24 (3.30)	10.61 (3.01)	0.62
MMSE (/30 ^§^)	21.62 (4.60)	27.94 (1.66)	-5.87^∗∗∗^
**Semantic tasks**			
Oral picture naming (/140)	40.33 (25.46)	125.00 (7.82)	-14.46^∗∗∗^
Picture associative matching (/70)	52.05 (8.65)	66.67 (2.28)	-7.45^∗∗∗^
Word associate matching (/70)	50.95 (9.77)	67.17 (1.54)	-7.50^∗∗∗^
**Non-semantic tasks**			
Calculation (/7)	6.38 (0.97)	6.44 (0.70)	-0.23
Rey-O Recall (/36)	10.43 (8.26)	17.06 (6.07)	-2.81^∗∗^

*^†^21 observations from 17 patients; ^‡^mean value (standard deviation); ^§^number of items; MMSE, Mini-Mental State Examination. ^∗^*p* < 0.05, ^∗∗^*p* < 0.01, ^∗∗∗^*p* < 0.001.*

### Behavioral Data Collection

Each subject’s semantic and non-semantic processing abilities were evaluated (**Table 1**), and each subject was individually tested over multiple sessions in a quiet room with rests allowed upon request.

#### Assessments for Semantic Ability

This ability was assessed using three tasks that share common semantic processing components but vary in their modalities of input and output (see a similar approach in Jefferies et al., 2008; Han et al., 2013). These tasks were run on a PC computer using the DMDX program (Forster and Forster, 2003). The presentation order of items in each task was randomized and was identical across subjects.

##### Oral picture naming

Participants were visually presented 140 color pictures, including 20 pictures from each of seven categories, namely, animals, tools, common artifacts, fruits and vegetables, large non-manipulable objects, faces and actions. They were required to correctly verbalize the name of the picture. Their responses were recorded using a digital voice recorder and were then manually transcribed off-line.

##### Picture associative matching

This task had the same format as the Pyramids and Palm Trees Test (Howard and Patterson, 1992). It included 70 items with ten items from each of the seven categories in the oral picture naming task. Each item consisted of three colored pictures. Participants were instructed to judge which of the two bottom pictures (e.g., tadpole or lion) was more semantically related to the top picture (e.g., frog).

##### Word associative matching

This task was identical to the picture associative matching task except that the pictures were replaced with their written names.

#### Assessments for Non-semantic Abilities

To investigate whether the neural bases we found are specific to semantic processing or general cognitive processes, two types of non-semantic abilities, namely, number processing and episodic memory were measured. They were the **number calculation task**, which included seven number calculation questions, and the **Rey-Osterrieth recall test** (Rey, 1941; Osterrieth, 1944).

### Behavioral Data Preprocessing

#### Raw Scores of Each Task

The semantic and the calculation tasks were scored based on the first complete response, whereas a common scoring procedure was used to score the Rey–Osterrieth recall test.

#### Corrected Scores of Each Task

Because the patient group revealed considerable variation in demographic properties (e.g., age, gender, and education), their raw scores may not meaningfully reflect the degree of deficits. To control for the influence of these individual characteristics, we adopted a standardization method proposed by Crawford and Garthwaite (2006) in which patients’ behavioral scores were corrected by considering the performance distribution of the healthy controls and transforming each patient’s raw score into a standardized *t* score (for details, see Han et al., 2013; Bi et al., 2015).

#### Semantic Composite Scores for Three Semantic Tasks

To obtain a measure that could more precisely reflect the severity of the semantic deficits of patients, we calculated a semantic composite score by averaging the *z*-transformed scores of the corrected *t* scores for each semantic task (see a similar method in Wei et al., 2012; Han et al., 2013; Bi et al., 2015). The composite score of each patient was treated as the semantic performance in the analyses.

### Imaging Data Acquisition

The SD patients and healthy control subjects were scanned using a 3T Siemens scanner at Huashan Hospital in Shanghai. The following three types of images were collected.

#### Structural T1 MRI Images

A T1-weighted 3D MP-RAGE sequence was scanned along the sagittal plane using the following parameters: repetition time (TR) = 2300 ms, echo time (TE) = 2.98 ms, flip angle = 9°, matrix size = 240 × 256, field of view (FOV) = 240 mm × 256 mm, slice number = 192 slices, slice thickness = 1 mm, and voxel size = 1 mm × 1 mm × 1 mm.

#### Resting-State fMRI Images

A functional echo-planar imaging (EPI) sequence was scanned on the transverse plane: TR = 2000 ms, TE = 35 ms, flip angle = 90°, matrix size = 64 × 64, FOV = 256 mm × 256 mm, slice number = 33 slices, slice thickness = 4 mm, and voxel size = 4 mm × 4 mm × 4 mm. During scanning, participants were instructed to close their eyes, remain still, and not think about anything systematically or fall asleep. The scan lasted for 400 s, and 200 volumes were acquired.

#### Diffusion-Weighted Images

The diffusion-weighted images were acquired with the transverse plane: 40 diffusion-weighting directions, TR = 8500 ms, TE = 87 ms, flip angle = 90°, matrix size = 128 × 128, FOV = 230 mm × 230 mm, slice number = 42 slices, slice thickness = 3 mm, and voxel size = 1.8 mm × 1.8 mm × 3 mm.

### Imaging Data Preprocessing

#### Structural T1 MRI Data

The data were skull-stripped and segmented into gray matter, white matter and cerebrospinal fluid with default resolution 1.5 mm × 1.5 mm × 1.5 mm using the VBM8 toolbox^1^ with the Statistical Parametric Mapping software (SPM8)^2^. Gray matter images were further normalized into Montreal Neurological Institute (MNI) space, modulated, smoothed with an 8-mm full-width-at-half-maximum (FWHM) Gaussian kernel and resampled into a 3 mm × 3 mm × 3 mm resolution.

#### Resting-State fMRI Data

These data were analyzed using SPM8 and Data Processing Assistant for Resting-State fMRI Advanced Edition (DPARSFA) (Yan and Zang, 2010). For each participant, the first ten functional volumes were discarded to ensure equilibrated magnetization and the adaptation of the participants. The remaining 190 volumes underwent slice timing and head motion correction. Two patient observations were discarded due to excessive head motion (>2.5 mm or >2.5° in maximum movement). To normalize functional images, the structural T1 image of each subject was co-registered to the realigned mean functional image. The transformation parameter of coregistration and segmentation was then used to register the functional images to the MNI space, with a resampling resolution of 3 mm × 3 mm × 3 mm. To reduce spatial noise, the functional images underwent spatial smoothing with a 4-mm FWHM Gaussian kernel. The linear trend of the time courses was removed, and a band-pass filter of 0.01–0.10 Hz was applied to reduce low-frequency drifts and high-frequency noise (Biswal et al., 1995; Lowe et al., 1998). Finally, six motion parameters and the time-series of white matter and cerebrospinal fluid signals were regressed out from the time course of each voxel. The residuals were used for the following resting-state functional connectivity analysis. The mean fractional amplitude of the low-frequency fluctuations (mfALFF) (Zang et al., 2007) for each voxel was calculated.

#### Diffusion-Weighted Imaging Data

The diffusion-weighted imaging data of each participant were first compiled into a single 4D image and preprocessed using the PANDA pipeline (Cui et al., 2013).^3^ The procedure included (1) using the BET to remove the skull; (2) using the eddy correct tool to correct for eddy current distortion; and (3) building diffusion tensor models.

The preprocessing analyses were conducted to obtain the following indices for each gray matter voxel: (1) the gray matter volume (GMV) value (derived from T1 image), which reflected the cortical volume of gray matter (Good et al., 2001); (2) the time course of each voxel (from rsfMRI), which reflected the BOLD signal fluctuation and was used later to calculate the functional connectivity; and (3) the mean fractional amplitude of low-frequency fluctuations (mfALFF, from rsfMRI), which reflected the intensity of functional physiological signals (Biswal et al., 1995) and later served as a confounding covariate in later validating analysis.

### Identifying SD-Semantic-Related Regions in the Brain Network

To find the regions associated with SD patients’ semantic deficits in the atrophic network of SD (Seeley et al., 2009; Zhou et al., 2012; Guo et al., 2013; La Joie et al., 2014), we first identified the SD-cortical atrophic network and the SD-functionally disconnected regions in the atrophic network.

#### The SD-Atrophic Network

A voxel-wise comparison of the GMV values between the SD patients and the healthy controls was conducted on each voxel of the whole brain. The clusters surviving the threshold of *p* < 0.05 AlphaSim correction (i.e., *p* < 0.05, cluster size >301 voxels) were masked as the atrophic regions of SD. AlphaSim stimulation was implemented using the new version of AFNI. Estimation of FWHM was based on the residual image of statistical model.

#### The Disconnected Region in the SD-Atrophic Network

For each participant, we first computed the FC strength of each pair of gray matter voxels in the SD-atrophic network, i.e., the Pearson correlation coefficient (*r*) between the rsfMRI signal intensity time courses of the two voxels. An effective FC was defined as having an *r* value >0.10 (Dai et al., 2014). We then calculated the degree centrality value of each voxel, i.e., the total number of effective FCs connecting to the voxel in the atrophic network (Zuo et al., 2012). The degree values of all voxels of a subject were further standardized as *z*-scores (Buckner et al., 2009). Finally, we obtained the functionally disconnected region of SD by comparing the standardized *z*-scores of degree centrality of each voxel between the two subject groups (AlphaSim corrected *p* < 0.05; single voxel *p* < 0.05, cluster size >103 voxels).

#### The SD-Semantic-Related Regions

We first extracted the seed for each SD-disconnected region. i.e., a 3 mm radius sphere whose center was at the peak of the region. The seed excluded the voxels outside the SD-atrophic mask. Then, correlation was calculated between the semantic composite scores and the mean *z*-degree values of the voxels within the seed across SD subjects. A region reaching the significance level (*p* < 0.05) might be considered as semantically related to SD.

### The SD-Semantic Functional Connections of the SD-Semantic-Related Regions

To explore how the SD-semantic-related regions function in concert with other regions for semantic processing, we performed a voxel-based correlation analysis between the seeded FCs of the SD-related regions with the semantic composite scores.

#### Identifying the SD-Semantic FCs

For each SD-semantic-related region observed herein, we calculated a standardized *z*-score of the FC between its seed and each remaining voxel in the SD-atrophic network. The *z* scores of each FC were correlated with the semantic composite scores of SD patients. The FCs surviving the AlphaSim corrected *p* < 0.001 (single voxel *p* < 0.05; cluster size >189 voxels) were treated as SD-semantic FCs.

#### Validating the Effects of the SD-Semantic FCs

To confirm that the effects of the observed SD-semantic relevant FCs were not driven by potential confounding factors, we performed again the semantic performance-FC strength correlation analyses additionally partialling out the influence of the following four variables: (1) the total GMV (summing the GMV values of all voxels in the whole brain), which was used to control for the overall severity of brain damage; (2) the structural signal intensity of the seed (summing the GMV values of all voxels in the 3 mm spherical seed) and (3) the functional signal intensity of the seed (summing the mfALFF values of all voxels in the seed), which were used to control for the influence of the node in the network; (4) the times of each patient observation (coding the first, second and third observations as 1, 2, and 3, respectively), which was used to control for practice or fatigue effects or the collinearity due to multiple repeated tests. To further exclude the influence of observation times, we correlated the semantic performance with the FC strength, excluding the follow-up data, i.e., only in the first observations of 17 subjects.

#### Testing the Functional Specificity of the SD-Semantic FCs

To investigate whether the observed SD-semantic FCs were specific to semantic processing or to general cognitive processing, we examined whether the intensity of each FC was correlated with the scores of two non-semantic tasks (corrected *t*-scores for number calculation and episodic memory tasks) and whether the effects of the FC-semantic correlations remained significant after covarying non-semantic task performances.

### Structural Basis of SD-Semantic Functional Connections

To elucidate whether the observed SD-semantic FCs had white matter fiber bases, we first tracked fiber for each SD-semantic FC in healthy subjects. Then, the measures of integrity of the obtained tracts were correlated with semantic performance across SD patients.

#### Constructing White Matter Tracts for Each SD-Semantic FC in Healthy Subjects

To examine the structural foundation of the semantic correlated FCs between the SD-semantic core regions and the observed clusters, we constructed fibers of each subject in native space using PANDA software (Cui et al., 2013). Firstly, for each white matter voxel, we obtained two complementary indices, namely, the fractional anisotropy (FA) value and the local diffusion homogeneity (LDH) value, which reflected the degree of myelination and microstructural coherence of the white matter, respectively (Gong, 2013). They were later used in fiber tracking and correlation analysis. Then, for each healthy subject, deterministic fiber tracking was performed using the FACT tracking algorithm (Mori et al., 1999) between the SD-semantic-related region and the clusters. Specifically, fiber tracking was terminated when the angle between two consecutive orientations was greater than a given threshold (45°) or when the FA value was less than a given threshold (0.15). Given that the outcome of tractography is affected by the initial position of the seed points within the voxel (Cheng et al., 2012), 100 seeds were randomly selected within each voxel to avoid biases from initial seed positioning. If fiber seeding within a voxel in the SD-semantic-related region succeeded in fiber tracking to one cluster ROI, that is, terminating in any voxel in that region, the cluster was considered to be connected to the SD-semantic-related region by this tracking path and with the passing voxels identified. Therefore, for each node pair of each subject, we obtained a tract mask connecting the two nodes that contained all passing voxels on any of the tracking paths between these two nodes. This tract mask is referred to as the tract between the two nodes for this subject.

For each pair of node masks, every tract of each subject was projected to the voxels in the native diffusion space, resulting in a voxel-based binary map. The binary map was further transformed to the MNI space. The tract maps of all subjects for this node pair were overlaid to generate a count map in which the value of each voxel represented the number of subjects with fibers on them. The count map was thresholded with *n* >3 (more than 20% of subjects) to obtain the tract mask.

#### Investigating Characteristics of the Integrity of White Matter Tracts in SD Patients

We first extracted the white matter integrity values of the tract for each subject, that is, the mean FA value and mean LDH value of all voxels on the tract mask. The two values were then compared between the two subject groups and correlated with semantic composite scores across SD individuals.

#### Validating the Effects of the SD-Semantic Structural Connectivity and Testing Its Functional Specificity

The methods were identical to those employed for the above FCs except that the SD-semantic FCs were replaced with SD-semantic structural connections.

## Results

### Neuropsychological Profiles

**Table 1** displays the background information and behavioral performance of the SD patients and the healthy control subjects. Compared with healthy subjects, the SD group exhibited profound deficits in semantic tasks (oral picture naming: *p* < 10^-15^; picture associative matching: *p* < 10^-7^; word associative matching: *p* < 10^-7^) and episodic memory task (*p* < 0.008). However, their number calculation ability was intact (*p* >0.80) (**Table 1**).

### SD-Semantic-Related Regions in Atrophic Network

**FIGURE 1:**
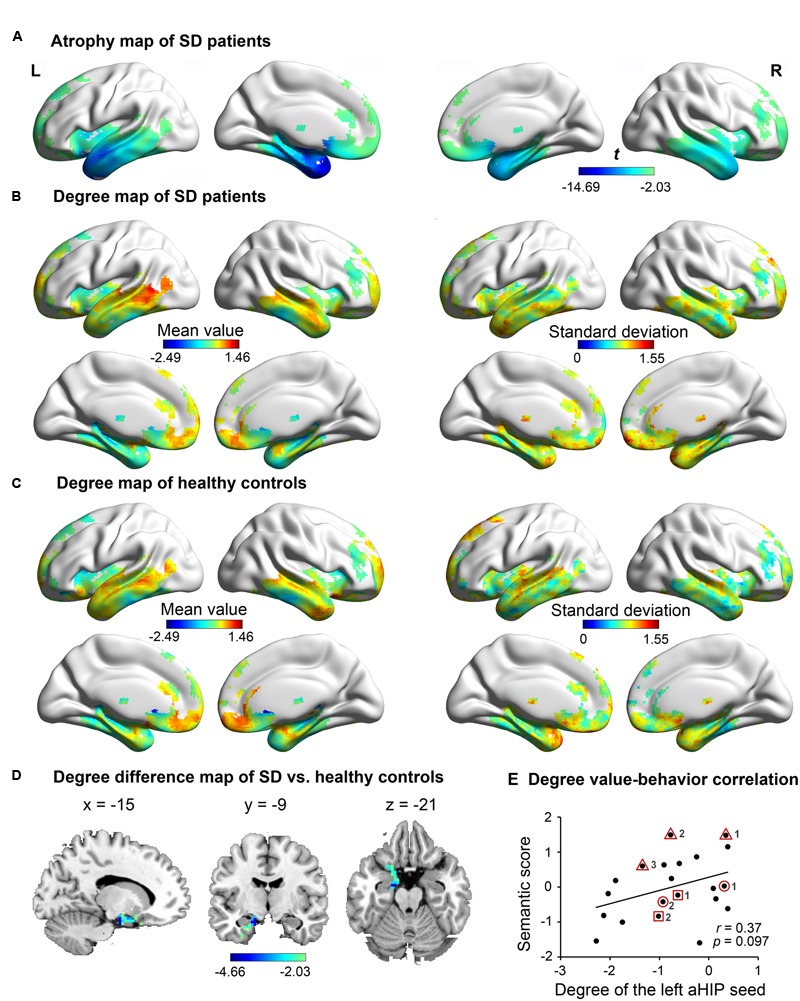
**The atrophy and degree-centrality maps in subjects.** In the atrophy map of SD **(A)**, the degree centrality value of the left anterior hippocampus (aHIP) **(D)** was significantly lower in SD patients **(B)** relative to healthy controls **(C)** (AlphaSim corrected *p* < 0.05); **(E)** correlation of the left aHIP-seeded degree-centrality values with semantic composite scores in SD. [the geometric figures (circle, square, and triangle) and numbers circling the dots show the three patients with multiple observations and their observation times, respectively].

**Figure 1A** illustrates the atrophic cortices of SD patients. The most severe atrophy occurred at the bilateral anterior temporal lobes and extended into the posterior temporal lobes, insula and ventral frontal lobes. Such an atrophy pattern of SD is highly consistent with the literature (Rosen et al., 2002; Desgranges et al., 2007; Yang et al., 2015). The distributions of degree centrality values between SD patients (**Figure 1B**) and healthy adults (**Figure 1C**) were similar in the SD-atrophic network except in the left aHIP, in which the values were significantly lower in SD patients (**Figure 1D**; AlphaSim corrected *p* < 0.05; MNI coordinates of cluster peak: -15, -9, -21; cluster size = 106 voxels). The distribution suggests that the left aHIP was disconnected in the brain networks of SD patients. Moreover, the degree centrality values of the disconnected region were marginally significantly correlated with semantic composite scores in SD (*r* = 0.37, *p* = 0.097; **Figure 1E**). Briefly, we observed that the left aHIP was an SD-disconnected and SD-semantic-related core region.

### SD-Semantic Functional Connections Connecting the Left aHIP

**FIGURE 2:**
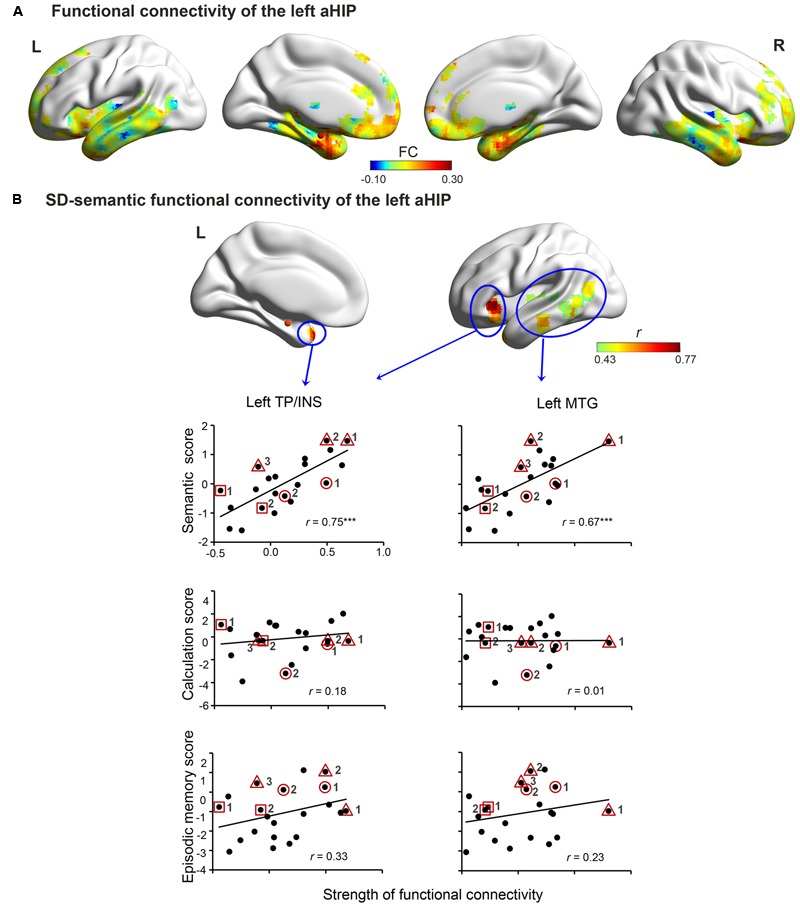
**Left aHIP-seeded functional connectivity in the SD brain.** The connectivity includes raw connectivity **(A)** and SD-semantic deficit-relevant connectivity **(B)** (AlphaSim corrected *p* < 0.001). In **(B)**, row 1 illustrates the correlated connectivity brain map. Rows 2, 3, and 4 present the correlation diagrams of connectivity strength with semantic scores, number calculation scores, and episodic memory scores, respectively. The red spheres denote the left aHIP seed. ^∗∗∗^*p* < 0.001. Full names of the abbreviations are provided in **Table 2**. The meaning of the geometric figures and numbers circling the dots see **Figure 1**.

In the SD-atrophic mask, the left aHIP seed was strongly functionally connected with the bilateral anterior temporal lobes, bilateral orbitalis of inferior frontal lobe, medial prefrontal gyrus and the right aHIP (**Figure 2A**). The FC *z*-scores between this seed and two regions significantly correlated with SD patients’ semantic composite scores (AlphaSim corrected *p* < 0.001; **Figure 2B** and **Table 2**). The regions included the left temporal pole/insula (left TP/INS; *r* = 0.75, *p* < 0.0001; peak coordinates: -33, 24, -3; cluster size = 5427 mm^3^) and the left middle temporal gyrus (left MTG; *r* = 0.67, *p* < 0.001; peak coordinates: -48, -15, 6; cluster size = 8370 mm^3^).

**Table 2:** Correlation coefficients between the integrity index of functional or structural connectivity of the left aHIP and the index of behavioral deficits in SD patients.

Connectivity type	Region connecting with the left aHIP	Connectivity integrity index	Correlation with semantic performance						Correlation with non-semantic task performance	
			Raw correlation	Correlation with the following variable as covariate					Number calculation	Episodic memory
				Total gray-matter volume	Structural signal intensity of the seed	Functional signal intensity of the seed	Covariation of multiple observations	All the four variables		
FC	Left TP/INS	FC intensity	0.75^∗∗∗^	0.70^∗∗∗^	0.64^∗∗^	0.75^∗∗∗^	0.77^∗∗∗^	0.64^∗∗^	0.18	0.33
	Left MTG	FC intensity	0.67^∗∗∗^	0.65^∗∗^	0.45^∗^	0.67^∗∗^	0.69^∗∗∗^	0.51^∗^	0.01	0.23
SC	Left TP/INS	FA value	-0.20	-0.34	-0.29	-0.21	-0.16	-0.39	-0.24	-0.30
		LDH value	-0.52^∗^	-0.48^∗^	-0.44^#^	-0.51^∗^	-0.51^∗^	-0.42^#^	0.20	-0.11

*^#^*p* < 0.10, ^∗^*p* < 0.05, ^∗∗^*p* < 0.01, ^∗∗∗^*p* < 0.001. aHIP, anterior hippocampus; FA, fractional anisotropy; FC, functional connectivity; INS, insula; LDH, local diffusion homogeneity; MTG, middle temporal gyrus; SC, structural connectivity; TP, temporal pole.*

More importantly, the observed effects of the two FCs held well even when we individually or totally partialled out the influence of four confounding factors (total GMV, GMV of the left aHIP seed, functional signal intensity of the left aHIP seed, and the observation times of each patient) (partial *r* = 0.45 to 0.77, *p*-values < 0.05; **Table 2**). Of note, these associations remained significant when we conducted the correlation analysis in the first 17 observations of the patients (*r* = 0.71–0.78, *p*-values < 0.01).

The two *z*FCs were not significantly correlated with the corrected *t*-scores of the two non-semantic tasks (number calculation: *r* = 0.01–0.18, *p*-values >0.42; episodic memory task: *r* = 0.23–0.33, *p-*values >0.14; **Figure 2B** and **Table 2**). However, the *z*FC scores were still significantly correlated with the semantic composite scores when the two non-semantic task performances were covaried (partial *r* = 0.60–0.74, *p*-values < 0.01). These results indicate that the FCs were specifically attributable to semantic deterioration of SD.

### SD-Semantic Structural Connections

**FIGURE 3:**
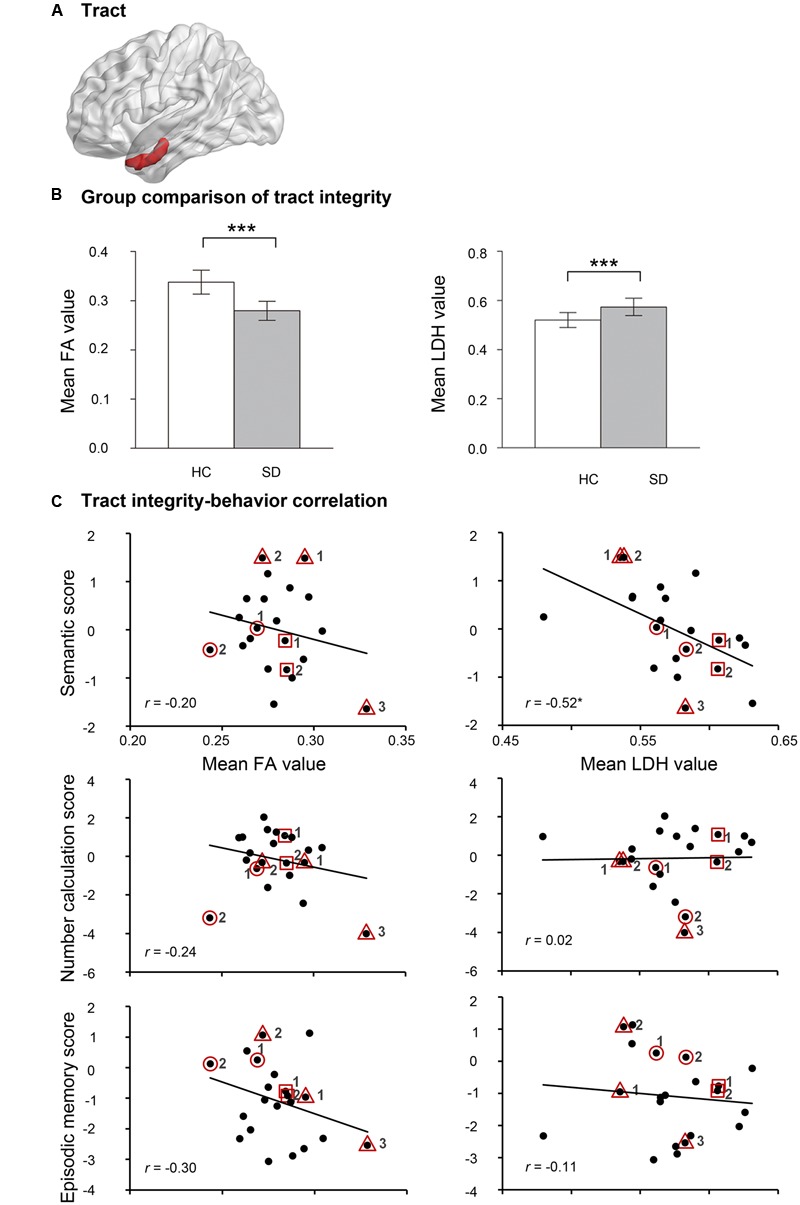
**The results of self-obtained white-matter tract connecting the left aHIP to the left TP/INS. (A)** Shematic of the self-obtained white-matter tract. **(B)** Group comparison of FA and LDH in healthy controls and semantic dementia. **(C)** Correlation between FA and LDH with semantic scores, number calculation scores and episodic memory scores in patients with semantic dementia. ^∗^*p* < 0.05, ^∗∗∗^*p* < 0.001. Full names of the abbreviations are provided in **Table 2**. The meaning of the geometric figures and numbers circling the dots see **Figure 1**.

To elucidate whether the two observed FCs had neuroanatomical bases, deterministic fiber tracking was first run between the two endpoints of each FC in healthy subjects. Only the left aHIP-left TP/INS FC was connected by a white matter fiber tract (cluster size = 576 mm^3^; **Figure 3A**). Relative to healthy controls, SD patients had significantly lower FA values (*t* = -8.40, *p* < 10^-9^) and higher LDH values (*t* = 4.98, *p* < 0.00002) on this tract (**Figure 3B**), indicating the damage to this tract in SD patients.

Moreover, one of the two integrity indices of this tract presented significant correlation with semantic composite scores in the SD group (LDH: *r* = -0.52, *p* < 0.02; FA: *r* = -0.20, *p* = 0.37; **Figure 3C**). This pattern was well preserved, with four confounding factors as covariates (LDH: partial *r* = -0.51 to -0.44, *p*-values < 0.051; FA: partial *r* = -0.34 to -0.16, *p*-values = 0.14–0.51; see **Table 2**). Moreover, the effects held well (LDH: *r* = -0.43, *p* = 0.08; FA: *r* = 0.21, *p* = 0.42) when the correlation analyses were conducted in the 17 first observations.

FA and LDH values of the left aHIP-left TP/INS tract did not correlate with the corrected *t* scores of the two non-semantic tasks (*r* = -0.30 or 0.24, *p-*values >0.19; **Figure 3C** and **Table 2**). However, the correlation between LDH values and semantic composite scores remained significant, partialling out non-semantic task performance (*p-*values < 0.02; **Table 2**). These results indicate that the tract might be primarily involved in semantic processing.

## Discussion

To identify the brain network devoted to the semantic deficits of SD, we correlated the semantic scores with the graph-theoretical network indices of the cortical regions and the functional/structural connectivity of SD patients. We found that the left anterior hippocampus (aHIP) was not only the SD-disconnected region but also the SD-semantic-related region. The strength of the functional connectivity between this region and two other regions, left TP/INS and left MTG, was associated with the semantic performance of the patients. Moreover, the left aHIP-left TP/INS connection presented a corresponding white matter anatomical basis. The observed effects of the functional and structural connectivity could not be fully accounted for by some potential confounding variables. In addition, no correlation was observed between these connections and performances on number calculation and episodic memory processing tasks.

### SD-Semantic-Related Core Region

The present study revealed that the left aHIP was a critical region whose disconnection was associated with the semantic disorders of SD. This result is highly consistent with a recent study emphasizing the importance of the aHIP in the disease-targeted network of SD (La Joie et al., 2014). They found that the resting-state functional connectivity of left aHIP in the normal brain mirrored the atrophy patterns of SD patients. Other studies also attempted to claim that this region contributes to SD-semantic deficits. However, most of those studies only focused on this region and did not investigate the role of its disconnections in the brain network (e.g., Chan et al., 2001; Mion et al., 2010). Though Agosta et al. (2014) found the disconnections of this region in the SD network, our study further confirmed that the disconnections of the left aHIP caused semantic disruptions of SD. The aHIP might serves a convergence center that binds multimodal semantic features into one coherent representation and forms concepts. Damage to this hub results in the semantic processing disruption of SD.

One recent study (Ding et al., 2016) highlighted the crucial role of the left fusiform gyrus (FFG) in SD-semantic deficits when the authors considered the GMV of local cortices. It is possible that the left HIP and FFG are both critical to semantic deficits of SD but they correspond to center of network connection and local regional functions, respectively. Another possibility is that the cerebral atrophy of SD does not cause the synchronous change between brain function and structure. The functional change-semantic deficits correlation results in findings in the left HIP, whereas the structural change in the left FFG.

### SD-Semantic Functional Connectivity

#### The Left aHIP-Left TP/INS Connection

The left aHIP intrinsically functionally connects to the left TP/INS in the resting-state human brain (La Joie et al., 2014; Qin et al., 2015). The TP is widely reported to be responsible for semantic knowledge representations based on studies of SD patients (Patterson et al., 2007), functional neuroimaging (Price et al., 2005) and repetitive transcranial magnetic stimulation (rTMS) (Pobric et al., 2010). The left insula was also found to be semantically related and responsible for emotional-affective semantics (Pulvermüller, 2013). Therefore, the left aHIP-left TP/INS connectivity facilitates the transport of formed concept knowledge to the storage of semantic representations and emotional-affective semantics. This connection damage results in the failure to form new semantic representations.

#### The Left aHIP-Left MTG Connection

The left MTG intrinsically connects with the left aHIP (Poppenk et al., 2008; Qin et al., 2015). The left MTG has been found to be involved in semantic processing (Binder et al., 2009) as it is activated in semantic tasks across stimuli input modalities (Vandenberghe et al., 1996; Stoeckel et al., 2003; Hickok and Poeppel, 2004; Maguire and Frith, 2004; Binder et al., 2009). Moreover, damage to the left MTG causes semantic and language comprehension deficits (Hart and Gordon, 1990; Kertesz et al., 1993; Dronkers et al., 2004). This region was suggested to be specific to semantic control (Hart and Gordon, 1990; Kertesz et al., 1993; Dronkers et al., 2004; Whitney et al., 2011, 2012). Stimulating the left posterior MTG selectively disrupted executively demanding semantic judgments. Thus, this connectivity damage led to the disruption of the choosing and retrieving of semantic knowledge.

### SD-Semantic Structural Connectivity

We observed that the left aHIP-left TP/INS were connected by white matter bundles and that its damage was associated with the semantic dysfunction in SD, thus demonstrating that this connectivity has functional and structural bases for semantic deficits in SD patients. Although both FA and LDH values reflected the integrity of a tract, on the left aHIP-left TP/INS tract, they presented a different pattern in SD patients. A possible interpretation is that LDH values may be more sensitive to microstructural coherence but be less sensitive to the degree of myelination than the FA values (Gong, 2013). When there is a disease-related axonal loss or demyelination associated with SD, the increased local coherence represents a compensatory mechanism.

### Limitations

This study has the following limitations: (1) The effects of TPs might not be well revealed due to the floor effect of their severe atrophy or susceptibility artifacts in fMRI data (Mion et al., 2010); (2) The connectivity outside the atrophic network of SD was not explored because it is not of principle interest in this research; (3) We included four follow-up observations in order to increase the number of observations which might raise some issues on validity of inferential statistics; (4) More specific semantic abilities (e.g., verbal vs. nonverbal semantics) were not investigated. Future studies with more fine-grained tasks are warranted; and (5) A limited number of indices were used to measure the topological attributes of the network.

## Conclusion

The left aHIP is the core semantic related region of SD, and functional disconnections between this region and two other regions (the left TP/INS and the left MTG) contribute to the semantic impairments of SD. Moreover, the left aHIP-left TP/INS connectivity has a neuroanatomical basis, and this connectivity is specific to semantic processing. These findings highlight the critical role of the left aHIP in semantic processing and provide direct evidence for the functional and anatomical framework of a SD-targeted semantic network.

## Author Contributions

QG and ZH designed the research; KC, QY, and YL performed the research; YC, KC, and JD analyzed the data; and YC, KC, YZ, QG, and ZH wrote the paper.

## Conflict of Interest Statement

The authors declare that the research was conducted in the absence of any commercial or financial relationships that could be construed as a potential conflict of interest. The reviewer FH and handling Editor declared their shared affiliation, and the handling Editor states that the process nevertheless met the standards of a fair and objective review.

## Footnotes

1 http://dbm.neuro.uni-jena.de/vbm8/

2 http://www.fil.ion.ucl.ac.uk/spm/

3 http://www.nitrc.org/projects/panda/
